# Supplementary material for: Tetrahedral DNA dendritic nanostructure-enhanced FISH for high-speed, sensitive spatial transcriptomics
Source: Nat Commun. 2025 Oct 20;16:9251. doi: 10.1038/s41467-025-64294-1 (PMC12537822; doi:10.1038/s41467-025-64294-1)
Supplement: Supplementary file 1 — Supplementary Information [file 41467_2025_64294_MOESM1_ESM.pdf]

Supplementary Information

for

Tetrahedral DNA dendritic nanostructure-enhanced FISH for high-speed, sensitive spatial transcriptomics

Yi-Fan Wang[1], Hua-Jie Chen[1], Zhong-Da He[1], Zhi-Gang Wang[2], Dai-Wen Pang[1], Shu-Lin Liu[1]*

[1]State Key Laboratory of Medicinal Chemical Biology, Frontiers Science Centre for New Organic Matter, Tianjin Key Laboratory of Biosensing and Molecular Recognition, Research Centre for Analytical Sciences, Frontiers Science Center for Cell Responses, College of Chemistry, Nankai University, Tianjin, P. R. China.

[2]School of Medicine, Nankai University, Tianjin, P. R. China.

*Email: shulin.liu@nankai.edu.cn

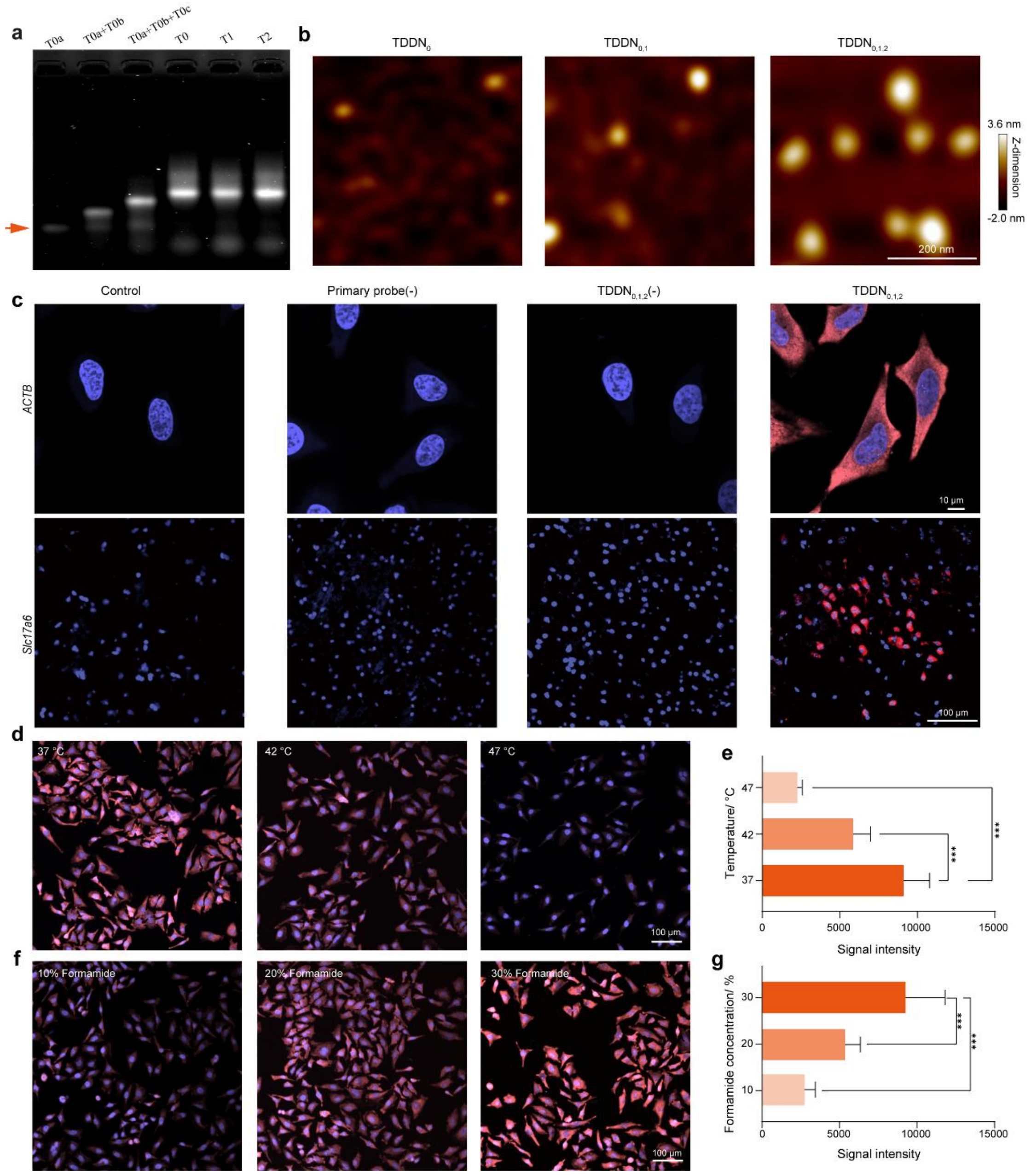

Supplementary Fig. 1 (a) Successful assembly of Tetrahedral DNA monomer (T0, T1, T2) demonstrated by gel electrophoretic analysis. Each DNA tetrahedron consists of 4 single strands. For example, T0 is composed by T0a, T0b, T0c and T0d, and the assemble process examined by gel electrophoretic analysis. The red arrow indicates the T0a bands. (b) Large-field atomic force microscopy (AFM) image of the TDDN. Scale bar, 200 nm. (c) The specificity of TDDN-FISH validated with multiple negative controls, including no treatment, no Primary probes, no TDDN in cells and frozen tissue sections. Scale bars, 10 μm and 100 μm. (d and e) Confocal imaging and quantitative analysis of *ACTB* mRNA in HeLa Cells labeled by TDDN-FISH at different primary probe hybridization temperatures. Scale bar, 100 μm. (f and g) Confocal imaging and quantitative analysis of *ACTB* mRNA in HeLa Cells labeled by TDDN-FISH at different formamide concentrations in the hybridization buffer. Scale bar, 100 μm. In panels e and g, the data are presented as mean ± s.d. (ns, not significant, $P > 0.05$; $^*P < 0.05$; $^{**}P < 0.01$; $^{***}P < 0.001$; unpaired two-tailed Student's t-test). Source data are provided as a Source Data file.

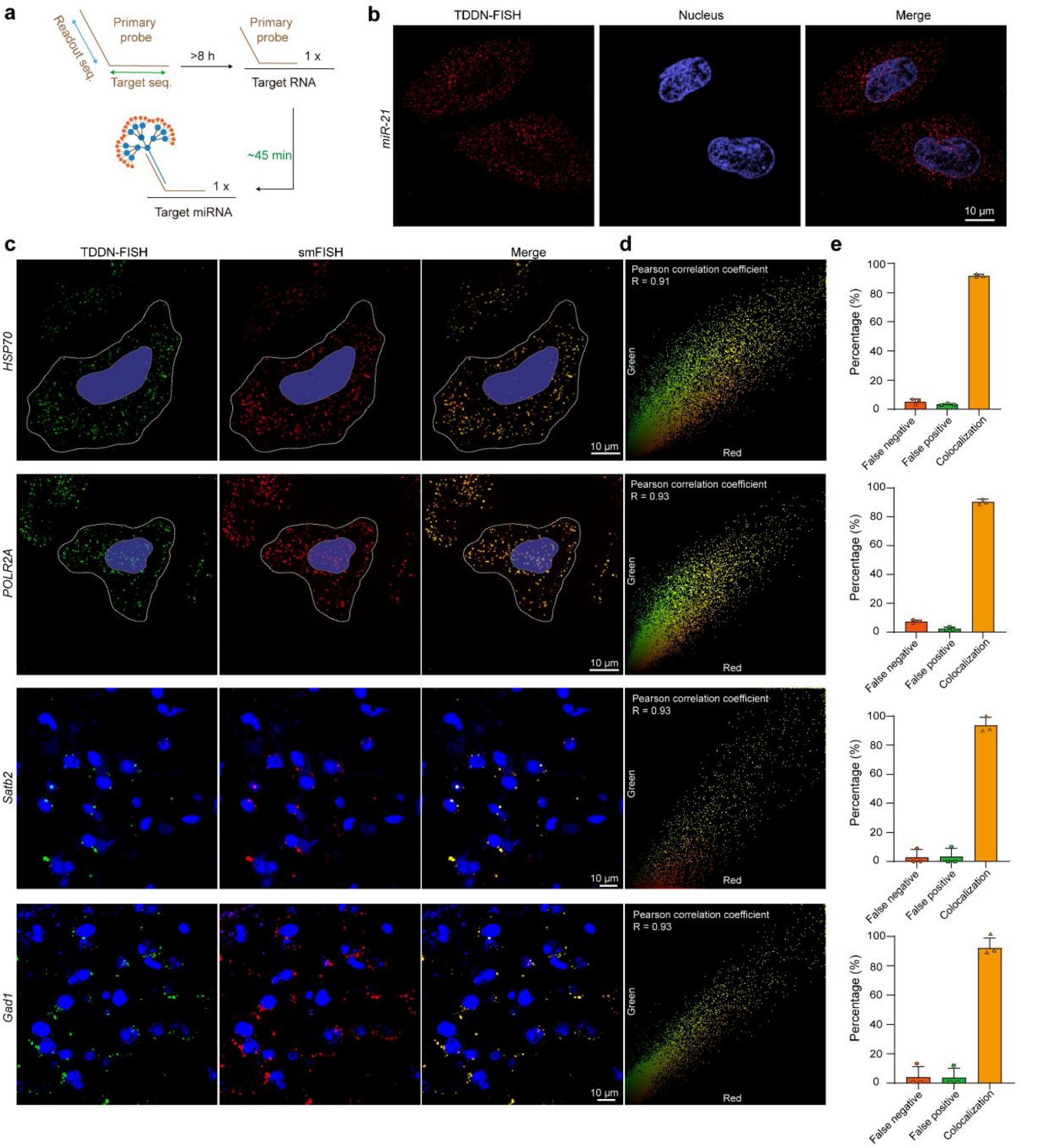

Supplementary Fig. 2 (a and b) Labeling procedure and dual-channel confocal images of miR-21 signals labelled by TDDN-FISH. (c) Dual-channel images of the RNA signals labeled by TDDN-FISH and smFISH. (d) 2D frequency scatterplots of the signals from TDDN-FISH and smFISH shown in (c). (e) Quantitative analysis of the colocalization signals (yellow), false positive signals (green) and false negative signals (red) shown in (d) (n = 3). In panels e, the data are presented as mean ± s.d. Source data are provided as a Source Data file.

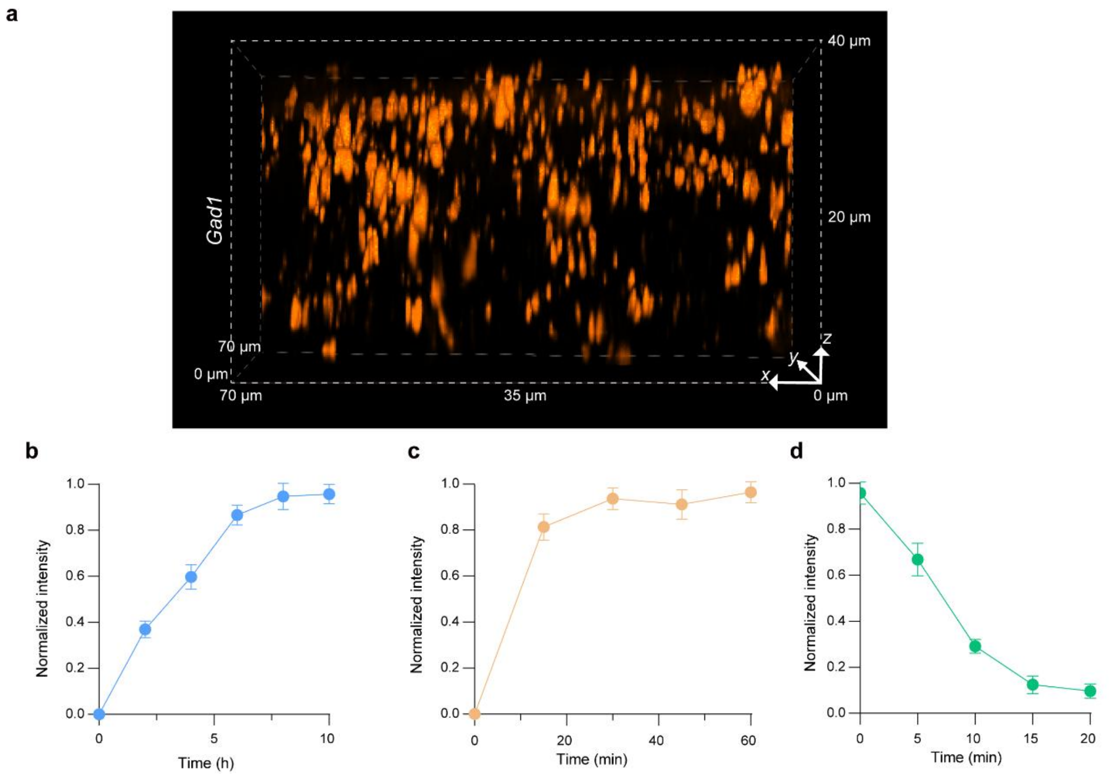

Supplementary Fig. 3 (a) TDDN-FISH imaging of *Gad1* mRNA in 40-μm-thick tissue sections. (b) Normalized mean intensity of each cell labeled with TDDN-FISH at different hybridization times of the primary probe. (c) Normalized mean intensity of each cell labeled with TDDN-FISH at different hybridization times of the TDDN probe. (d) Normalized mean intensity of each cell labeled with TDDN-FISH under different cleavage durations of TCEP(n = 3, biological replicates). Source data are provided as a Source Data file.

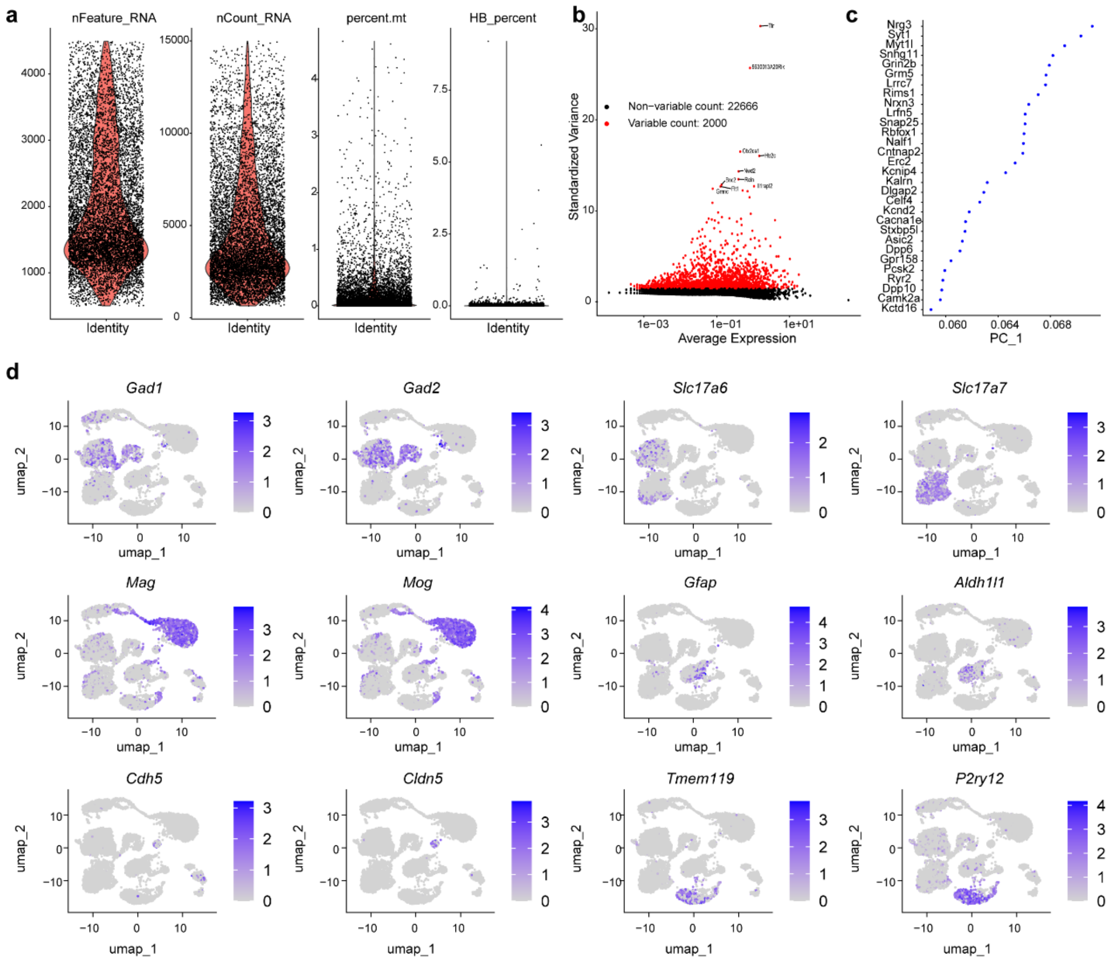

Supplementary Fig. 4 (a) Violin plot for quality control of scRNA-seq data. From left to right: number of detected genes per cell (nFeature_RNA), total UMI counts per cell (nCount_RNA), percentage of mitochondrial gene expression (percent.mt), and percentage of hemoglobin gene expression (HB_percent). (b) Scatter plot of highly variable genes. (c) Dimensionality reduction scatter plot of highly variable genes. (d) UMAP plot of marker genes for six selected cell types used for cell annotation.

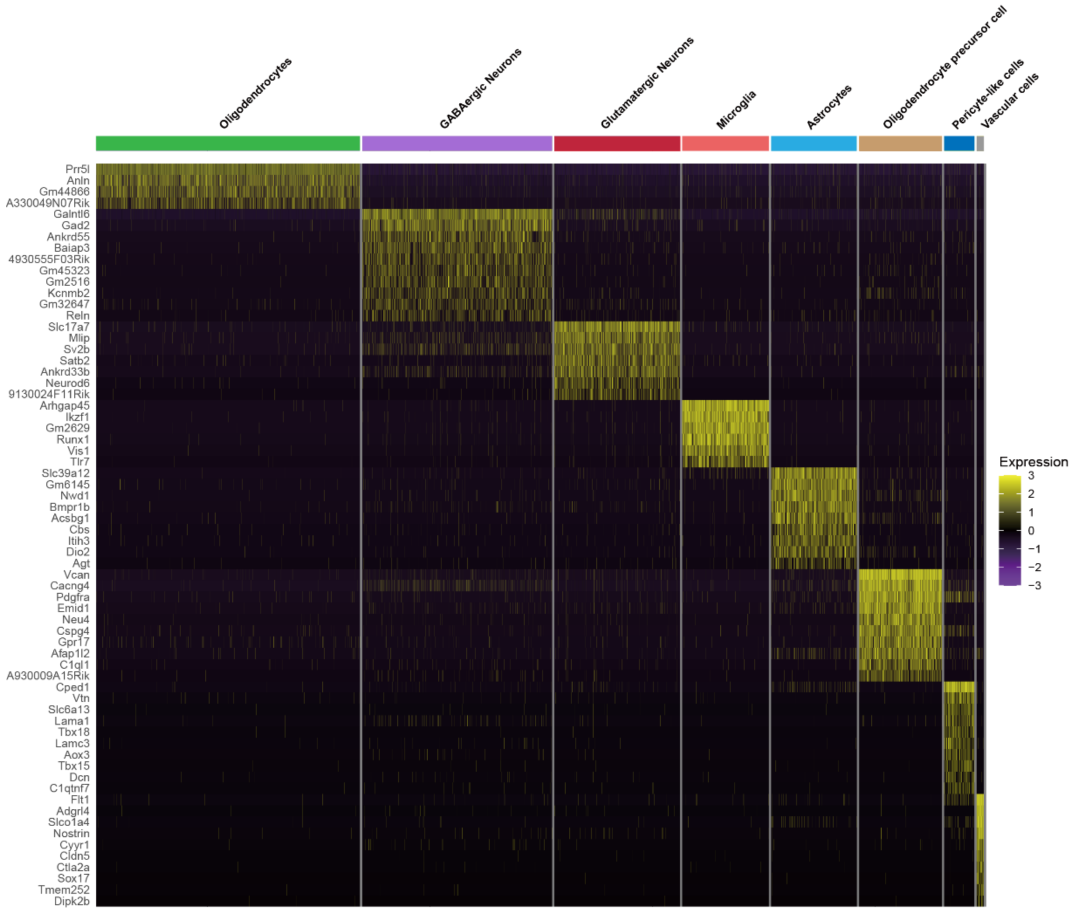

Supplementary Fig. 5 Heatmap of mouse brain scRNA-seq data.

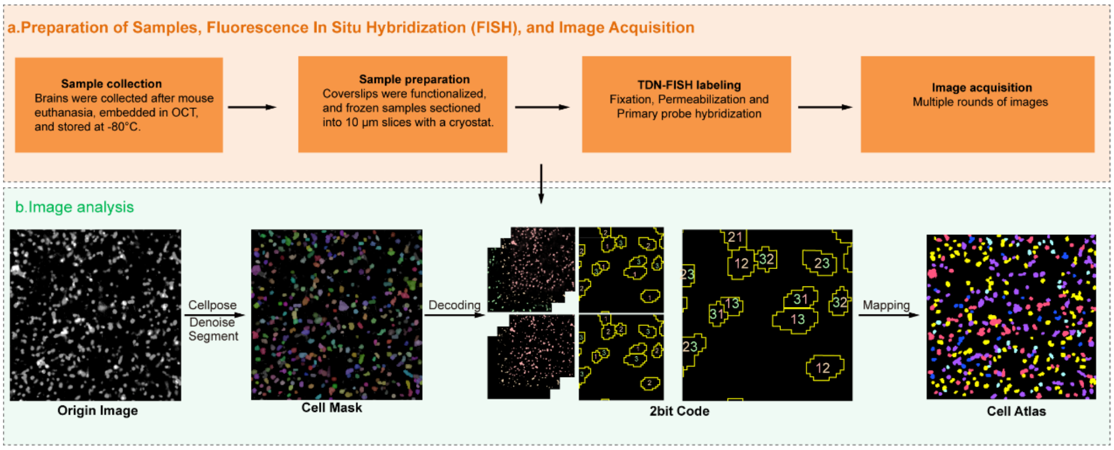

Supplementary Fig. 6 The workflow of TDDN-FISH imaging for frozen tissue sections includes two main stages: image acquisition and image analysis. In the image acquisition stage, the sample is first prepared by embedding brain tissue in optimal cutting temperature compound (OCT). and sectioning it into 10 μm slices. The tissue sections undergo permeabilization, fixation, and hybridization, followed by multi-round image acquisition. After image acquisition, the analysis begins with cell segmentation using Cellpose[13], which generates a mask for each cell. The cell masks are then matched with the second round of images, and each mask is assigned a color barcode. Finally, a cellular distribution map is constructed based on the color-coded masks.

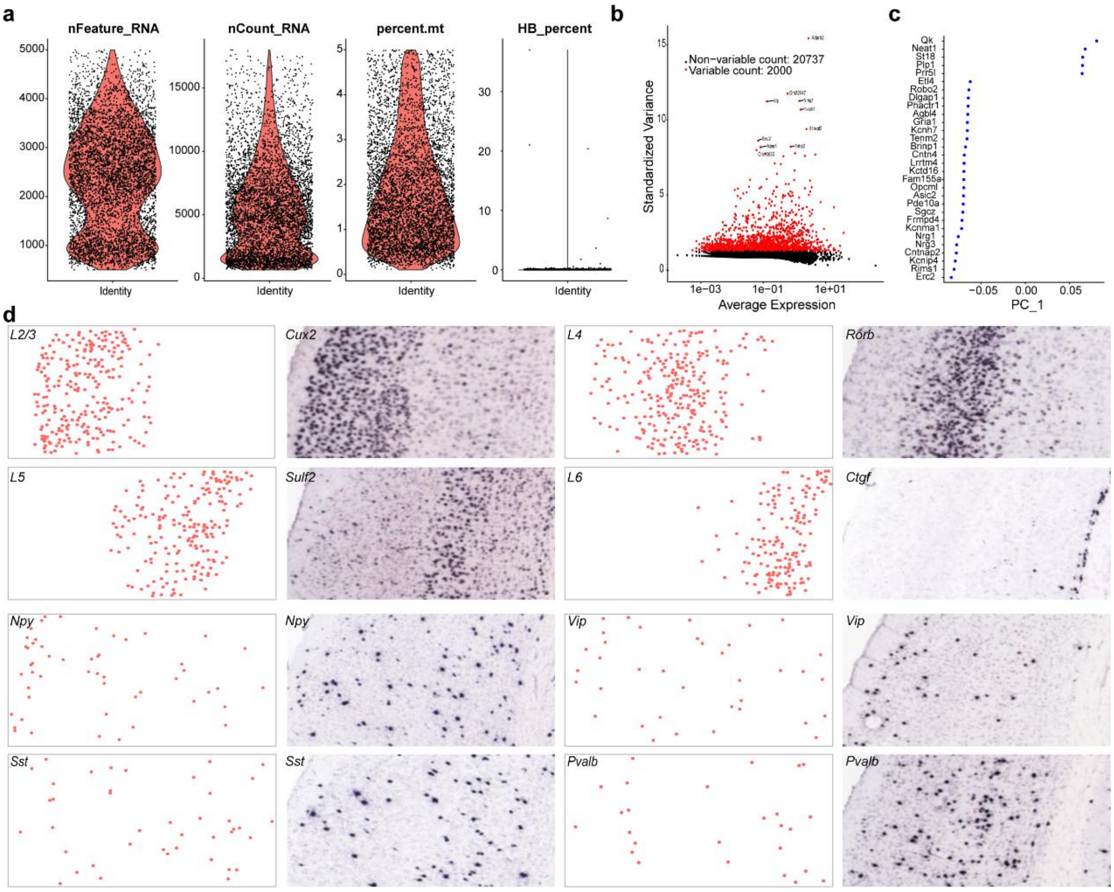

Supplementary Fig. 7 (a) Violin plot for quality control of mouse brain visual cortex scRNA-seq data. From left to right: number of detected genes per cell (nFeature_RNA), total UMI counts per cell (nCount_RNA), percentage of mitochondrial gene expression (percent.mt), and percentage of hemoglobin gene expression (HB_percent). (b) Scatter plot of highly variable genes. (c) Dimensionality reduction scatter plot of highly variable genes. (d) Validation of TDDN-FISH (Left) in situ images from Allen Institute of Brain Science (AIBS). (Right) TDDN-FISH-extracted cell distribution pattern reliably reproduced the spatial gene expression pattern from AIBS. L1 to L6 denote the six layers of the neocortex. Vip, Npy, Sst, and Pvalb represent four major types of inhibitory neurons.

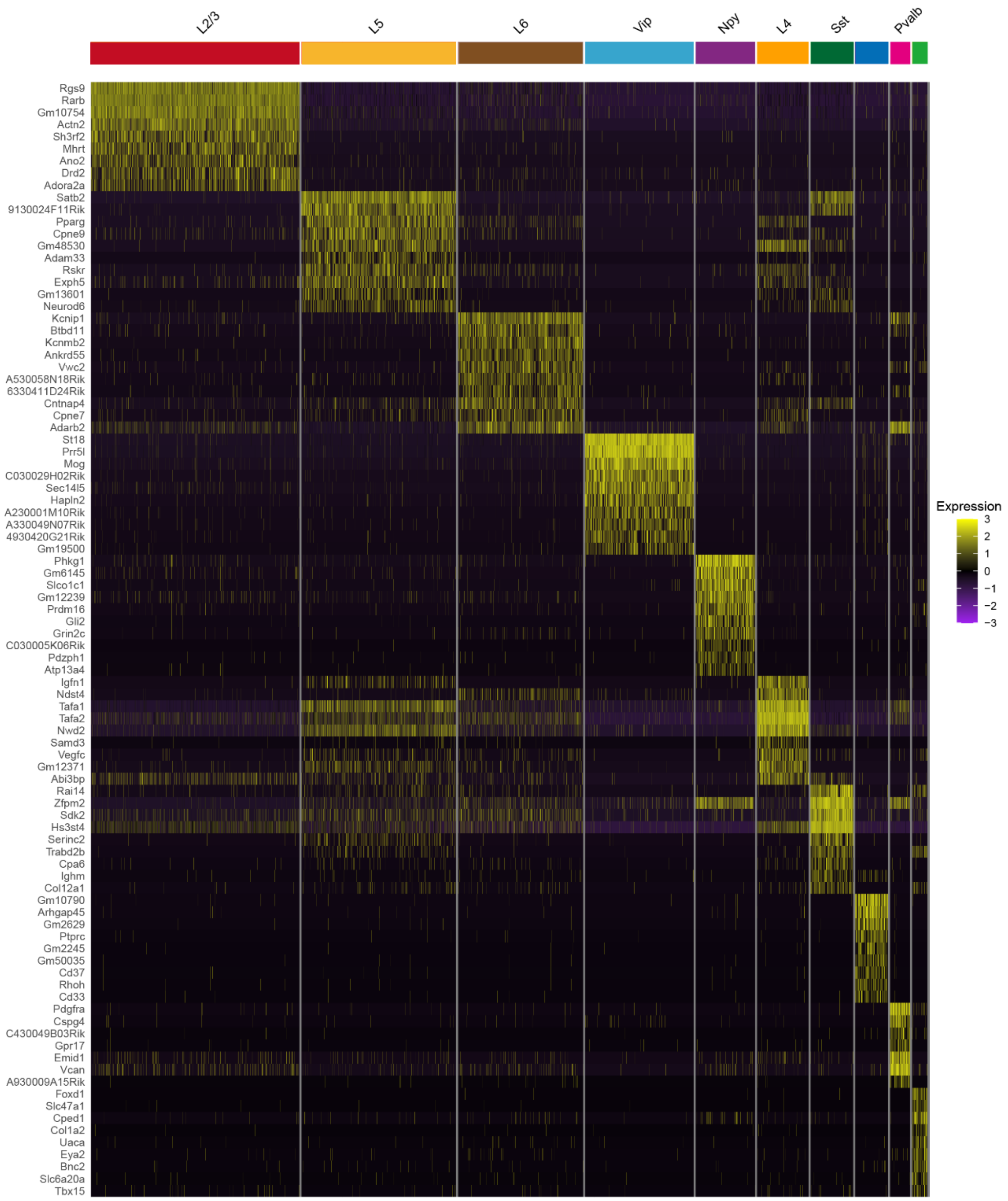

Supplementary Fig. 8 Heatmap of mouse visual cortex scRNA-seq data.

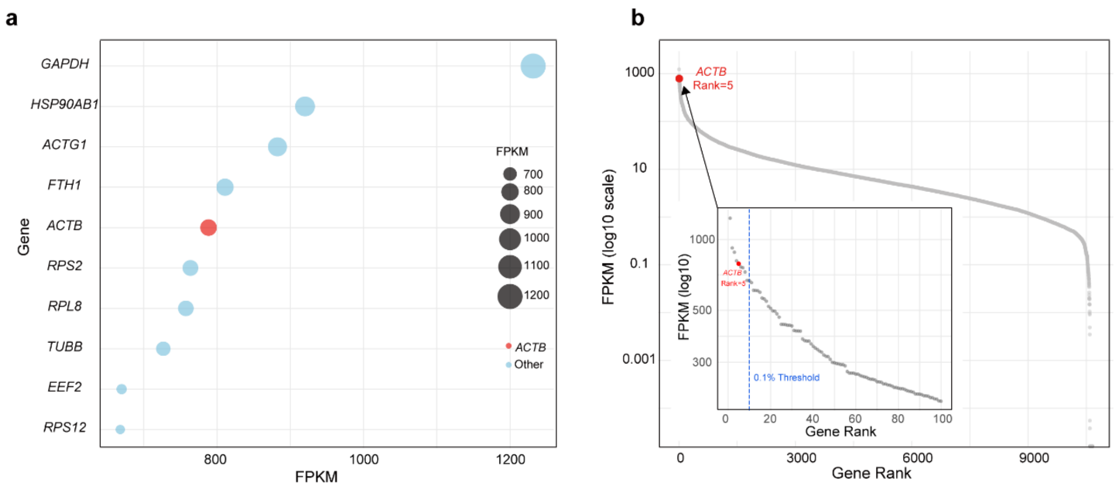

Supplementary Fig. 9 Genome-wide Expression Profiling and Ranking of HeLa Cells Based on FPKM Values. (a) Bubble plot of the top 10 highly expressed genes, with *ACTB* shown in red. Bubble size reflects FPKM value. (b) Gene expression ranking based on FPKM values from HeLa RNA-seq data. The red dot marks *ACTB* (Rank = 5), and the blue line indicates the top 0.1% expression threshold by expression rank.
