# Supplementary material for: Tetrahedral DNA dendritic nanostructure-enhanced FISH for high-speed, sensitive spatial transcriptomics
Source: Nat Commun. 2025 Oct 20;16:9251. doi: 10.1038/s41467-025-64294-1 (PMC12537822; doi:10.1038/s41467-025-64294-1)
Supplement: Supplementary file 2 — Reporting Summary [file 41467_2025_64294_MOESM2_ESM.pdf]

nature portfolio

Corresponding author(s): Shu-Lin Liu

Last updated by author(s): Sep 13, 2025

Reporting Summary

Nature Portfolio wishes to improve the reproducibility of the work that we publish. This form provides structure for consistency and transparency in reporting. For further information on Nature Portfolio policies, see our Editorial Policies and the Editorial Policy Checklist.

Statistics

For all statistical analyses, confirm that the following items are present in the figure legend, table legend, main text, or Methods section.

n/a	Confirmed	
[ ]	[x]	The exact sample size (*n*) for each experimental group/condition, given as a discrete number and unit of measurement
[ ]	[x]	A statement on whether measurements were taken from distinct samples or whether the same sample was measured repeatedly
[ ]	[x]	The statistical test(s) used AND whether they are one- or two-sided<br>*Only common tests should be described solely by name; describe more complex techniques in the Methods section.*
[x]	[ ]	A description of all covariates tested
[x]	[ ]	A description of any assumptions or corrections, such as tests of normality and adjustment for multiple comparisons
[ ]	[x]	A full description of the statistical parameters including central tendency (e.g. means) or other basic estimates (e.g. regression coefficient) AND variation (e.g. standard deviation) or associated estimates of uncertainty (e.g. confidence intervals)
[ ]	[x]	For null hypothesis testing, the test statistic (e.g. *F*, *t*, *r*) with confidence intervals, effect sizes, degrees of freedom and *P* value noted<br>*Give P values as exact values whenever suitable.*
[x]	[ ]	For Bayesian analysis, information on the choice of priors and Markov chain Monte Carlo settings
[x]	[ ]	For hierarchical and complex designs, identification of the appropriate level for tests and full reporting of outcomes
[ ]	[x]	Estimates of effect sizes (e.g. Cohen's *d*, Pearson's *r*), indicating how they were calculated

Our web collection on statistics for biologists contains articles on many of the points above.

Software and code

Policy information about availability of computer code

Data collection	The atomic force microscopy images were obtained using a Bruker Dimension Icon microscope. Confocal images were obtained with a Nikon Ti2 microscope. Images for agarose gel electrophoresis were taken using the Bio-Rad ChemiDoc imaging system. Fiji ImageJ software was used to quantify both microscopy images. Adobe illustrator 2020 software packages was used to assemble images into figures.
Data analysis	Statistical analysis was performed using GraphPad Prism 8.0. Statistical significance was determined by unpaired two-tailed Student's t-test and one-way analysis of variance (ANOVA) test.

For manuscripts utilizing custom algorithms or software that are central to the research but not yet described in published literature, software must be made available to editors and reviewers. We strongly encourage code deposition in a community repository (e.g. GitHub). See the Nature Portfolio guidelines for submitting code & software for further information.

Data

Policy information about availability of data

All manuscripts must include a data availability statement. This statement should provide the following information, where applicable:

- Accession codes, unique identifiers, or web links for publicly available datasets
- A description of any restrictions on data availability
- For clinical datasets or third party data, please ensure that the statement adheres to our policy

All other data supporting the findings of this study are available in the article and its Supplementary Information. All data are available from the corresponding authors upon request.

Research involving human participants, their data, or biological material

Policy information about studies with human participants or human data. See also policy information about sex, gender (identity/presentation), and sexual orientation and race, ethnicity and racism.

Reporting on sex and gender	No human research
Reporting on race, ethnicity, or other socially relevant groupings	No human research
Population characteristics	No human research
Recruitment	No human research
Ethics oversight	No human research

Note that full information on the approval of the study protocol must also be provided in the manuscript.

Field-specific reporting

Please select the one below that is the best fit for your research. If you are not sure, read the appropriate sections before making your selection.

[x] Life sciences   [ ] Behavioural & social sciences   [ ] Ecological, evolutionary & environmental sciences

For a reference copy of the document with all sections, see nature.com/documents/nr-reporting-summary-flat.pdf

Life sciences study design

All studies must disclose on these points even when the disclosure is negative.

Sample size	For the in vivo experiments, The sample sizes were indicated in the figure legends.
Data exclusions	No data were excluded from this manuscript.
Replication	The experimental repeat numbers are indicated in the figure legends.
Randomization	The mice used in the study were randomly allocated.
Blinding	Data acquisition was performed by investigators blinded to the groups.

Reporting for specific materials, systems and methods

We require information from authors about some types of materials, experimental systems and methods used in many studies. Here, indicate whether each material, system or method listed is relevant to your study. If you are not sure if a list item applies to your research, read the appropriate section before selecting a response.

Materials & experimental systems

n/a	Involved in the study	
[x]	[ ]	Antibodies
[ ]	[x]	Eukaryotic cell lines
[x]	[ ]	Palaeontology and archaeology
[ ]	[x]	Animals and other organisms
[x]	[ ]	Clinical data
[x]	[ ]	Dual use research of concern
[x]	[ ]	Plants

Methods

n/a	Involved in the study	
[x]	[ ]	ChIP-seq
[x]	[ ]	Flow cytometry
[x]	[ ]	MRI-based neuroimaging

Eukaryotic cell lines

Policy information about cell lines and Sex and Gender in Research

Cell line source(s)	Hela, A549 and MDCK cell lines were obtained from the Shanghai Cell Bank, Chinese Academy of Sciences.
Authentication	Hela, A549 and MDCK cell lines are commercially available and have been tested or authenticated by the manufacturers on the basis of standard techniques including morphology check, isoenzyme analysis and mycoplasma detection.
Mycoplasma contamination	Mycoplasma contamination was not detected.

Commonly misidentified lines (See ICLAC register)	No commonly misidentified cell line was used in this study.

Animals and other research organisms

Policy information about studies involving animals; ARRIVE guidelines recommended for reporting animal research, and Sex and Gender in Research

Laboratory animals	Six-week old female Balb/c mice were purchased from Beijing Vital River Laboratory Animal Technology Co. Ltd (Beijing, China). The mice were housed in specific-pathogen free conditions at 25°C and 40 – 60% relative humidity, with a 12-hour light/dark cycle. Food and water were available ad libitum.
Wild animals	No wild animal was involved in this study.
Reporting on sex	As the primary aim of this study was to demonstrate the feasibility of the method in animal samples, sex was not considered in the experimental design or analysis.
Field-collected samples	No field-collected sample was involved in this study.
Ethics oversight	All mouse studies were approved and performed iin accordance with the guidelines set by the Animal Care and Use Committee of Nankai University(SYXK (Jin) 2019-0003)

Note that full information on the approval of the study protocol must also be provided in the manuscript.

Plants

Seed stocks	No plants
Novel plant genotypes	No plants
Authentication	No plants
