# Supplementary material for: Tetrahedral DNA dendritic nanostructure-enhanced FISH for high-speed, sensitive spatial transcriptomics
Source: Nat Commun. 2025 Oct 20;16:9251. doi: 10.1038/s41467-025-64294-1 (PMC12537822; doi:10.1038/s41467-025-64294-1)
Supplement: Supplementary file 4 — Description of Additional Supplementary Files [file 41467_2025_64294_MOESM4_ESM.pdf]

Description of Additional Supplementary Files

Supplementary Data 1

Details of tetrahedral DNA sequence design, TDDN-FISH sequences, and detailed sequences of the probes used for smFISH and HCR.
